# Assessing Pharmacy Costs of Intravenous Push Controlled Substance Waste in Hospital-Based Areas: A Multi-Site Study

**DOI:** 10.3390/pharmacy13050121

**Published:** 2025-09-01

**Authors:** John Hertig, Les Louden, Blake Shay, Armando Soto, Thi Doan, Zach Gross

**Affiliations:** 1 Hertig Healthcare Advising, LLC, Indianapolis, IN 46220, USA; 2 Adjunct Faculty, Purdue University, West Lafayette, IN 47906, USA; 3 BayCare Health System, Tampa, FL 33607, USA; les.louden@baycare.org (L.L.); blake.shay@baycare.org (B.S.); armando.soto@baycare.org (A.S.); zach.gross@baycare.org (Z.G.)

**Keywords:** ready to administer, waste, intravenous, IV push, pharmacy, cost effectiveness

## Abstract

Intravenous push (IVP) administration of controlled substances in hospital settings presents operational challenges related to medication waste, documentation, and diversion risk. This multi-site observational study aimed to quantify the pharmacy workforce time and associated costs linked to IVP waste management across a 16-hospital health system in Southwest Florida. Data were collected from over 4400 controlled substance transactions involving fentanyl, midazolam, hydromorphone, morphine, ketamine, and lorazepam. Methods included automated transaction analysis, manual chart reviews, and software-based compliance case evaluations. Results indicated patterns of partial dose waste, particularly for midazolam (85.2%) and hydromorphone (78.8%), and identified opportunities where documentation efforts could be further optimized through automation. Manual review of 333 incidents required an average of 6 min and 43 s per case, extrapolating to over 496 h of quarterly pharmacy labor or nearly 1985 h annually. Software-based case reviews added another 32 h per quarter or 130 h annually. Additionally, waste receptacle systems incurred over USD 1.1 million in capital costs and USD 322,500 in annual maintenance, with technician labor contributing further operational burden. These findings underscore the resource demands of IVP waste management and support the need for standardized dosing, enhanced documentation workflows, and pharmacy-led interventions to improve efficiency and reduce diversion risk.

## 1. Introduction

Injectable medications are widely used across many health systems and are common root causes of medication errors. About one-third to one-half of such medication errors that occur with intravenous (IV) medications are preparation and administration errors [1]. These errors lead to more than one million hospitalizations annually, adding around USD 2.7–5.1 billion of medical costs to the US healthcare system [1]. Controlled substances are associated with medication errors and carry unique risks, including drug diversion. Meanwhile, health system policies and procedures for handling and disposing of controlled substances vary. Controlled substances, including fentanyl, hydromorphone, and morphine, are associated with particularly high abuse and diversion risk, and therefore, proper disposal of these agents is best practice. The Drug Enforcement Agency (DEA) estimates that prescription drug diversion in the United States is a USD 25 billion-a-year industry [2].

Bluesight™, a medication intelligence company, has reported diversion and controlled substance documentation data from over 900 clinical sites. This report confirms the highest variance in documentation rates with fentanyl, midazolam, hydromorphone, morphine, and lorazepam due to incorrect documentation (50%), issues with documentation timing (31%), and missing documentation (19%). Of these drugs, fentanyl is reported as the drug with the highest variances (24%), followed by midazolam (15%), hydromorphone (10%), morphine (7%), and lorazepam (6%) [3]. Depending on the patient care unit, the quantity and variety of controlled substances administered can create an administrative and regulatory burden on healthcare professionals. Policies requiring thorough documentation, checks and balances, and audits necessitate an institutional investment of time and resources. There are significant costs associated with proper controlled substance disposal, management, and regulatory compliance. In the context of the current opioid crisis, and given the high abuse potential of fentanyl, hydromorphone, and morphine, it is imperative that waste is minimized and waste procedures are followed to ensure safe disposal of controlled substances. Previous research has been conducted in operating rooms demonstrating significant costs associated with both product waste and the workforce time associated with compliance [4,5,6,7,8].

A prospective multicenter study in Italy focused specifically on observing this phenomenon in intensive care, emergency areas, and operating room settings. The study observed that the estimated yearly waste amounted to 139,531 syringes for a total estimated financial cost of EUR 78,060 (USD 92,569) [4]. Furthermore, previous studies by Hertig et al. found significant product waste (PW) and nurse and anesthesia workforce time waste (WTW) associated with controlled substances in procedural care areas, with an estimated WTW of USD 56,557 over a span of 10 days [9,10]. However, limited data exist for pharmacy workforce time waste and the resulting implications on overall waste minimization practices in inpatient hospital settings. This information is critical to support the expanded adoption of improved waste compliance processes for controlled substances. While previous research has focused on clinical nursing and anesthesia areas, research is needed to better understand the pharmacy-specific financial and workforce impacts associated with drug waste in these healthcare settings. The purpose of the study was to quantify pharmacy workforce time and costs association with IV controlled substance products in inpatient hospital units. Specific data on medication volume will help to evaluate pharmacy involvement during the medication use process. The primary aim was to quantify the total pharmacy workforce time and costs associated with administering ketamine, midazolam, fentanyl, hydromorphone, morphine, and lorazepam via the IVP route.

## 2. Materials and Methods

This study was conducted at a large 16-hospital health system, consisting of over 3899 licensed beds, in Southwest Florida, including facilities such as St. Joseph’s Hospital, St. Joseph’s Women’s & Children’s Hospital, and Morton Plant Hospital. Given the high abuse potential of controlled substances, it is imperative that waste procedures are followed to ensure proper waste disposal. St. Joseph’s Hospital Main Campus is a 615-bed tertiary care facility that includes a 100-bed Emergency Department, 26-bed Medical and Surgical ICUs, a 26-bed Cardiac Care Unit, and other specialized units. Additionally, the facility includes 32 beds dedicated to Pre-Op and PACU recovery. The campus also encompasses St. Joseph’s Women’s Hospital and St. Joseph’s Children’s Hospital, which contribute 100 OB-GYN and Neonatal ICU beds and 219 Pediatric beds, respectively. This integrated layout reflects the typical format followed across BayCare hospitals, though unit composition and bed counts vary depending on facility size, service lines, and regional demand.

The study objective was evaluated through the quantification of pharmacy workforce time defined as WTW associated with (1) process incidents that require pharmaceutical waste (PW) and (2) total time required to reconcile various reported PW incidents. These were measured through continuous reporting of waste and administration documentation. The secondary aim of this study was to analyze workflow practices related to evaluating costs for waste disposal practices and WTW required to maintain waste disposal requirements.

This observational study was conducted across a wide range of inpatient and procedural care environments within a large, multi-hospital health system. Clinical units included high-acuity areas such as Intensive Care Units (ICUs), Emergency Departments (EDs), and Post-Anesthesia Care Units (PACUs), as well as general inpatient medical–surgical floors; pediatric and neonatal intensive care units (PICUs and NICUs); and specialized procedural areas like the Operating Room (OR), Cardiac Catheterization Lab (Cath Lab), and Endoscopy Suites. Maternal–child health units (e.g., Labor and Delivery and Postpartum), behavioral health units, and outpatient infusion and dialysis centers were also represented. This diversity of settings strengthens the generalizability of the findings by capturing the varied workflows, documentation practices, and medication use patterns that influence pharmacy workforce time waste across the continuum of hospital care.

**Figure 1:**
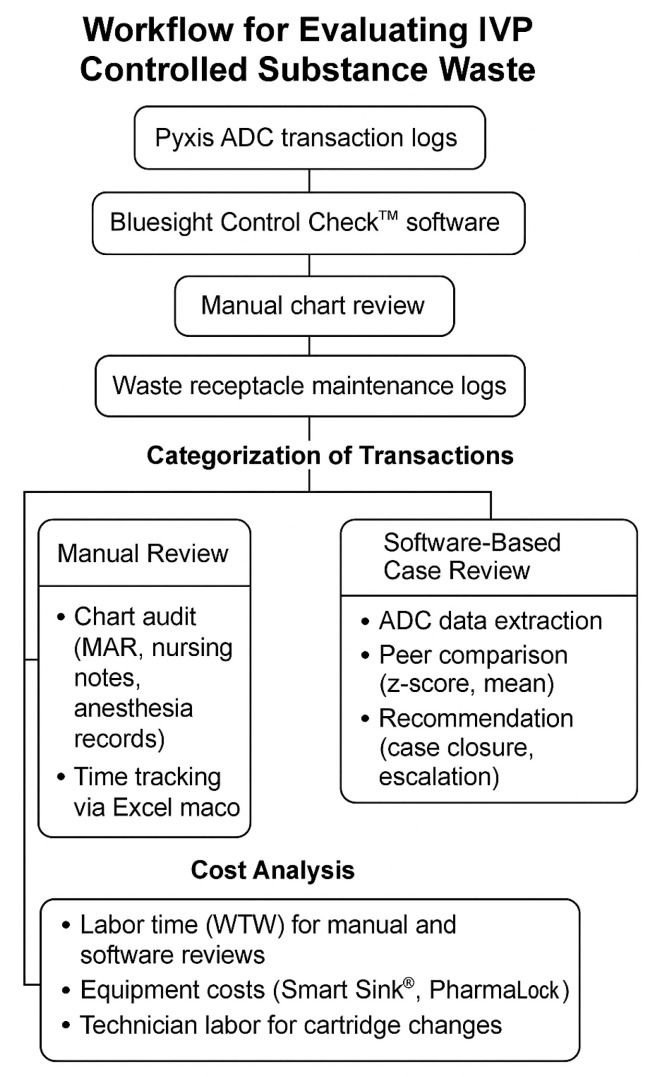
Workflow for evaluating IVP controlled substance waste.

All hospital-based areas were identified and evaluated using controlled substance volume data, with similar mechanisms for tracking controlled substance waste. In settings where automated systems were used to manage controlled substances, a pharmacy workflow time study design and a subset of continuous direct observation time motion studies were employed. A data collection tool developed in a previous study by Hertig et al. [9] was used to capture medication type, waste amount, activity timestamps, total time, and the number of incidents reported. Once automated methods and processes were observed, a standardized process was synthesized for data analysis. Consistent with previous studies, descriptive statistics were used. Data measures such as number of assessments, workforce waste, and incident reporting were reported for each drug (ketamine, midazolam, fentanyl, hydromorphone, morphine, and lorazepam) individually and in aggregate. For continuous variables, mean, standard deviation, and median (with minimum and maximum values) were calculated. For categorical variables, frequency tables including counts and percentages were generated. A visual representation of the decision-making workflow used to evaluate IVP controlled substance waste is provided in Figure 1.

Bluesight Control Check™ (formerly Protenus^®^) analyzed data from BD Pyxis^TM^ medication dispensing stations, focusing on controlled substance incident-related transactions that were returned to stock, disposed of, or unreconciled. A transaction was considered unreconciled when the dispensed amount did not match the documented quantity for administration, waste, or return. The analysis included incident-related transactions involving IV push fentanyl, hydromorphone, midazolam, morphine, ketamine, and lorazepam, recorded from October to December 2024 across 16 hospital sites. Transactions were excluded if the dispensed amount was zero or if the unreconciled amount was zero or negative. Remaining unreconciled transactions were categorized as:

Likely Waste: Partial administration documented but remainder unaccounted for.

Likely Administration: Partial waste documented but remainder unaccounted for.

Both: No documentation of waste or administration or totals that did not reconcile with the dispensed amount.

The total number of incident-related transactions was calculated for each medication, along with a half-vial analysis to identify the most commonly wasted vial type. Transactions were classified as “half-wasted” when the documented waste equaled exactly half of the standard vial size. Importantly, while “both” transactions may reflect incomplete data capture, they do not confirm improper handling. These often originate from procedural areas—such as Catheterization Labs, Radiology, and other settings—where alternative documentation systems or paper charting may be used. Because these systems may not be fully integrated with the electronic health record platforms that feed into Bluesight Control Check™, they contribute to a high volume of transactions with missing or partial documentation. Similarly, “likely waste” and “likely administration” transactions also represent incident-related events that may be documented elsewhere but require manual validation. These classifications arise when automated systems cannot definitively determine the outcome of a medication transaction, necessitating manual human review to confirm whether the medication was administered, wasted, or otherwise handled. This presents a significant operational challenge, as all such transactions, regardless of category, require manual review to validate medication handling.

“Manual Review of Likely Waste, Administration or Both”

Bluesight Control Check™ was used to extract detailed data for each incident, including the incident ID; date; associated automated dispensing cabinet (ADC); and records of medication handling actions (e.g., dispensed, completed, and disposed). To validate these data, a single research team member conducted a comprehensive manual review of patient charts. This review was carried out over a six-week period in March and April 2025.

A standardized process was developed to guide the review and ensure all potentially unverified components—such as missing documentation for administration, waste, or both—were thoroughly assessed. This included examining nursing notes, anesthesia records, operative and procedural documentation, flow sheets, and the Medication Administration Record (MAR). The goal was to cross-reference and confirm the accuracy of the medication handling data reported in Bluesight Control Check™. To support consistent time tracking, an Excel-based database was created with an integrated macro. This tool enabled the reviewer to log start and stop times using clickable buttons, which automatically applied timestamps to the relevant fields, streamlining the documentation of each unverified waste evaluation.

“Incident-Related Transactions Case Review”

When incidents such as delayed waste documentation, unreconciled drug transactions (e.g., missing waste or administration records), delayed administrations, or other discrepancies are identified, Bluesight Control Check™ software uses machine learning algorithms to generate cases for further evaluation. These cases are flagged based on patterns of concern and categorized as “Very Suspicious”, “Suspicious”, or unclassified, using statistical comparisons such as mean and z-score analyses relative to peer activity within similar ADC usage groups. For this study, a designated Pharmacy Diversion Analyst was responsible for tracking timestamps throughout the case evaluation process. The analyst reviewed cases that included at least one unreconciled medication, along with any other incident-related events. These cases occurred over a one-year period, from May 2024 to May 2025. To support accurate time tracking, a Microsoft^®^ Excel (Microsoft Excel, Version 2506, 2025; Microsoft Corporation, Redmond, WA, USA) database was developed with an integrated macro that allowed the analyst to record start and stop times using clickable buttons. These actions automatically applied timestamps to the relevant data fields, streamlining the documentation process during case evaluations. Based on this workflow, three core steps were identified in the compliance review process:

1. Pulling ADC Data—Retrieving up to 30 days of automated dispensing cabinet (ADC) transaction data relevant to the case timeframe.
2. Reviewing Data—Analyzing key metrics such as waste transactions, frequency of vends per product, and daily usage patterns. This step also involved comparing the subject’s activity to peer benchmarks across ADCs and hospital units to identify trends or anomalies.
3. Providing a Recommendation—Summarizing findings and offering a recommendation for the next steps. These recommendations support hospital leadership in determining appropriate follow-up actions, which may include case closure, referral to a nurse manager, or escalation to the diversion response team (DRT).

This structured approach ensures consistency in case evaluation and supports timely, data-driven decision-making in the controlled substance diversion review process.

“IV Push Waste Receptable Costs and Waste Receptable WTW”

To evaluate the operational costs associated with IV push waste receptacle systems, two analyses were conducted. First, the capital and ongoing costs of waste receptacle products (e.g., Cactus Smart Sink^®^ and Pharma Lock^®^ Controlled Substance Waste Management System), based on the number of BD Pyxis^TM^ locations at each facility, was calculated. For each site, the number of installed waste receptacle units was determined and associated startup and maintenance fees were extracted from vendor contracts to estimate total system costs.

Pharmacy-associated WTW was related to upkeep and maintenance of these systems. This included technician labor required for routine tasks such as replacing batteries and cartridges. A sample size was determined to evaluate the average time required for these maintenance activities, with scheduled observations conducted at Morton Plan Hospital, where cartridge changes occur biannually (as also practiced at St. Joseph’s Hospital (SJH)). Time data collected from these observations was used to estimate labor costs associated with WTW across facilities. The research was approved as exempt from full instructional review board review by the BayCare Health System Institutional Review Board (IRB FWA00008336).

## 3. Results

A total of 4432 controlled substance transactions were analyzed for fentanyl, hydromorphone, midazolam, morphine, ketamine, and lorazepam. Each transaction was categorized based on documentation patterns into one of three groups: “Likely Waste”, “Likely Administration”, or “Both” (which includes transactions with incomplete or ambiguous documentation). As shown in Table 1A, fentanyl had the highest number of total transactions (1871), followed by midazolam (1217) and hydromorphone (528). Notably, midazolam had the highest proportion of transactions categorized as “Likely Administration” (367), while fentanyl had the most evenly distributed documentation across all three categories. Morphine and lorazepam also showed a high number of “Both” transactions, suggesting frequent gaps in documentation for these agents. Ketamine, while representing the smallest sample size (33 transactions), still demonstrated a notable proportion of transactions lacking clear documentation.

A secondary analysis focused on transactions where the dispensed amount matched the standard vial size, allowing for an evaluation of how often exactly half of the vial was documented as waste. This revealed consistent patterns of partial dose waste, particularly for midazolam (85.2%), hydromorphone (78.8%), and morphine (75.0%), suggesting opportunities for standardization in dosing or packaging to reduce waste. These findings are further detailed in Table 1B, which summarizes the frequency of partial dose waste.

A total of 333 randomly selected medication incidents were reviewed in this arm of the study, including 193 fentanyl, 57 hydromorphone, 26 midazolam, 46 morphine, 5 ketamine, and 6 lorazepam. The average pharmacy WTW per incident varied by medication, with fentanyl requiring 6:54 min, hydromorphone 7:12 min, midazolam 4:44 min, morphine 6:25 min, ketamine 6:31 min, and lorazepam 6:48 min, as detailed in Table 2A. Overall, the average WTW for all 333 incidents was 6:43 min per incident, amounting to a total of approximately 37 h, 36 min, and 39 s spent confirming the accuracy of medication handling data from Bluesight Control Check™. When these findings were extrapolated to the 4432 medication incidents from the fourth quarter (October–December), the total pharmacy workforce time waste was estimated to be 496 h, 8 min, and 16 s, or nearly 1985 h annually, as summarized in Table 2B.

A total of 35 medication cases were reviewed using the software compliance review process. These cases represent a subset of medication incidents that required further investigation and formal case creation. Each case underwent a structured three-step review process: pulling ADC data, reviewing the data, and providing a recommendation. On average, Step 1 (Pull ADC Data) required 3 min and 13 s, Step 2 (Review Data) took 4 min and 57 s, and Step 3 (Provide Recommendation) took 3 min and 20 s. The total average time per case was 11 min and 30 s. The standard deviation for each step was 28 s, 1 min and 24 s, and 26 s, respectively, with an overall standard deviation of 1 min and 17 s for total review time, as detailed in Table 3A. When extrapolated to the full set of 170 cases that were reviewed during the fourth quarter (October 1–31 December 2024) of 2024, the total pharmacy workforce time spent conducting software compliance reviews was approximately 32 h, 31 min per quarter, which equates to roughly 130 h and 4 min annually, as shown in Table 3B.

Table 4 outlines the list prices (as of 2022 and not including any discounts, etc.) associated with IV push waste receptacle systems, including setup and maintenance costs for both solid and liquid waste disposal. The Cactus Smart Sink^®^, priced at USD 1500 per unit, requires both solid and liquid cartridges, while the Cactus Pharma Lock^®^ OR sink, used specifically in anesthesia areas, costs USD 675 and requires only a liquid cartridge. Smart sinks were deployed across 15 hospital locations, totaling 503 units, and Pharma Lock^®^ OR units were installed in 284 anesthesia-related locations, resulting in a combined total of 787 waste receptacle units. Deployment varied by site size: small sites required fewer than 10 units, medium sites 20–60 units, and large sites over 100 units. Based on setup costs of USD 1750 per Med Station and USD 800 per Pharma Lock^®^ OR unit, the total estimated list price for equipment deployment was approximately USD 1,107,450.

These figures represent initial capital investments; however, it is important to note that ongoing maintenance costs, including cartridge replacements every six months and potential equipment servicing, are required to sustain long-term functionality and compliance. Cartridge replacements are needed every six months, with each cartridge costing USD 125. Given the replacement frequency and cartridge requirements of four cartridges annually per smart sink and two per Pharma Lock^®^ OR unit, the estimated annual maintenance list costs are approximately USD 322,500.

To estimate the labor costs associated with controlled substance waste handling across all hospital locations, a baseline hourly wage of USD 20 was applied for pharmacy technicians. At BayCare, approximately 750 waste receptacle units require technician involvement, consisting of 500 Cactus Smart Sink units (each with 2 cartridges) and 250 Pharma Lock units (each with 1 cartridge), totaling 1250 cartridge exchanges annually. Each cartridge exchange takes an average of 8.2 min of focused technician time. This time encompasses tasks such as exchanging solid and liquid waste cartridges.

While over half of the units are battery-powered, battery replacements were excluded from this analysis, as they are addressed on an ad hoc basis. Based on this methodology, the estimated pharmacy technician labor across all hospitals is approximately USD 854 per quarter, totaling USD 3416 annually. This corresponds to a total of 170 h and 50 min of technician time per year or approximately 1 day, 18 h, and 43 min per quarter dedicated to waste receptable cartridge exchanges. It is important to note that this estimate reflects uninterrupted, task-specific time. In practice, workflow interruptions and variations in facility layout such as the location of automated dispensing cabinets (ADCs) can significantly affect task duration.

## 4. Discussion

This multi-site observational study provides a comprehensive assessment of pharmacy WTW associated with IVP controlled substance waste in inpatient hospital settings. It uniquely quantifies the time and resource burden on pharmacy personnel across five key areas: automated incident-related transactions and avoidable waste, manual verification of incident-related transactions, software-based incident case review, waste receptacle system costs, and waste receptacle maintenance and technician labor.

The study evaluated controlled substance utilization across a diverse range of hospital settings. As expected, high transaction volumes were observed, particularly in areas where medication administration may not have been fully captured in the electronic health record systems integrated with Bluesight Control Check™. These include procedural environments such as Catheterization Labs, Radiology, and other specialized units where alternative documentation systems or even paper charting may be used for medication reconciliation. The variability in documentation platforms across these settings contributed to discrepancies in recorded administration data. The findings highlight the scale of avoidable waste, with over 4400 transactions analyzed and fentanyl accounting for the highest volume of unreconciled and disposed units. Notably, a substantial proportion of transactions involved the disposal of exactly half of a vial, particularly for midazolam (85.2%) and hydromorphone (78.8%), suggesting a systemic pattern of partial dose waste that may be amenable to packaging or dosing standardization interventions.

Further contextualizing the operational burden, the time required for manual review of 333 medication incidents averaged 6 min and 43 s per incident, translating to over 37 h of pharmacy labor for the reviewed samples and an estimated 496 h when extrapolated to all 4432 incidents from the fourth quarter of 2024. This reinforces the significant time investment required to ensure accurate documentation and regulatory compliance, particularly in the absence of automated reconciliation tools.

A more intensive review process involving 35 escalated cases required formal case creation and software-based compliance review. Each case underwent a structured three-step process pulling ADC data, reviewing documentation, and providing a recommendation, with an average total time of 11 min and 30 s per case. When extrapolated to the 170 cases reviewed during the study period, this equated to over 32 h of pharmacy labor. These findings suggest that, while escalated cases represent a smaller subset of total incidents, they demand a disproportionately higher time commitment due to their complexity and the need for multidisciplinary follow-up. It is important to note that the time analysis captured only the core review steps of data retrieval, documentation review, and recommendation. Additional time commitments, such as scheduling meetings, attending diversion response team discussions, and engaging with suspected team members, were not included in the measured workflow. These activities vary by hospital and pharmacy department, depending on internal protocols and the structure of diversion response procedures, and represent further unquantified demands on pharmacy resources.

The financial investment required to implement IV push waste receptacle systems across 15 hospital locations included a total of 787 units, 503 Cactus Smart Sink^®^ and 284 Pharma Lock^®^ OR units, resulting in an estimated capital list cost of USD 1.1 million. These systems require ongoing maintenance, primarily cartridge replacements every six months, contributing to an annual maintenance list cost of USD 322,500.

In addition to equipment costs, technician labor required to support these systems averaged 8.2 min per unit, totaling approximately 170 h of technician time and USD 3416 in annual labor costs. While battery-powered units were not included in this review, they represent an additional ad hoc maintenance consideration. These findings underscore the importance of accounting for both capital and operational demands when evaluating the long-term sustainability of waste receptacle systems.

Together, these results underscore the multifaceted nature of pharmacy WTW in the context of controlled substance waste. They also highlight opportunities for targeted interventions, such as optimizing vial sizes (matching products with practice), enhancing documentation systems, and leveraging automation to reduce manual workload. Importantly, the study provides a replicable framework for quantifying pharmacy labor costs associated with waste management, which can inform resource allocation and policy development across health systems. By including such a comprehensive cross-section of hospital units, the study provides a robust foundation for identifying system-wide opportunities to improve waste documentation, streamline pharmacy workflows, and reduce the risk of diversion.

Notably, while reviewing all incident-related transactions is more time-consuming than reviewing individual cases, this is expected. Diversion-focused software is designed to streamline the process by generating mostly accurate data and organizing it into manageable cases, making manual review by pharmacy personnel more efficient and targeted.

There is extensive evidence in the literature outlining the risks IVP carries in hospitals and health systems [11,12,13,14,15,16,17,18,19,20]. The data resulting from this study supplement previous cost and waste evidence, adding additional pharmacy-specific information to help inform health systems as to the total cost of waste when using IV push products. Institutions should perform their own holistic data analysis to help determine potential cost savings, waste, and other operational efficiencies, including safety assessments, based on their unique workflows and documentation systems [1,9,10,21].

Limitations of this study include the potential impact of seasonality on medication consumption patterns. For example, the extrapolation of incident data from a single quarter to estimate annual trends may not account for fluctuations due to seasonal illnesses such as influenza and COVID-19 or variations in elective surgery volumes throughout the year. Additionally, documentation discrepancies arising from non-integrated systems and manual charting may have led to underreporting or misclassification of waste events. The study also did not capture indirect time commitments such as meetings and follow-up actions related to diversion investigations, which may further contribute to pharmacy workloads. Additionally, the cost estimates presented in this study are based on specific brands and models of waste receptacle systems (e.g., Cactus Smart Sink^®^ and Pharma Lock^®^), and other technologies or vendors may yield different financial outcomes for institutions adopting alternative approaches. Finally, the generalizability of the findings may be limited to institutions with similar infrastructure and documentation practices.

## 5. Conclusions

This multi-site observational study demonstrates that pharmacy WTW associated with IVP controlled substance waste is both measurable and operationally significant. Across multiple review modalities, including automated transaction analysis, manual chart review, and software-based case evaluation, pharmacy personnel invested substantial time in documenting, verifying, and resolving waste-related incidents.

The average time per incident ranged from approximately 6 to 11 min, with extrapolated quarterly and annual burdens exceeding 496 and 1984 h for manual reviews and 32 and 130 h for escalated case evaluations. Additionally, the study identified consistent patterns of partial dose waste, particularly for midazolam, hydromorphone, and morphine, highlighting opportunities for standardization in dosing or packaging. Beyond time spent on direct case review, further unmeasured labor is required for scheduling, multidisciplinary meetings, and diversion response team activities, which vary by institution. The study also quantified the financial and labor costs associated with waste receptacle systems, adding another layer to the hidden operational burden of IVP waste management.

These findings underscore the need for health systems to invest in waste reduction strategies, such as standardized dosing, matching product availability with practice, improved documentation workflows, and integrated analytics platforms. By quantifying both the direct and indirect labor costs of waste management, this study supports the case for pharmacy-led interventions that enhance compliance, reduce diversion risk, and improve overall operational efficiency.

## Figures and Tables

**Table 1:** (**A**) Incident-related transactions. (**B**) Half-vial waste analysis.

**(A)**				
**Medication**	**Likely Waste**	**Likely** **Administration**	Both *	**Total Transactions**
Fentanyl	602	619	650	1871
Hydromorphone	150	115	263	528
Midazolam	70	367	780	1217
Morphine	46	65	332	443
Ketamine	9	9	15	33
Lorazepam	94	110	136	340
**(B)**				
**Medication**	**Vial Size**	**Eligible** Transactions **	Half Wasted Transactions ***	**% Half Wasted**
Fentanyl	100 mcg	1227	749	61.04%
Hydromorphone	1 mg	193	152	78.76%
Midazolam	2 mg	398	339	85.18%
Morphine	4 mg	44	33	75.00%
Ketamine	200 mg	20	5	25.00%
Lorazepam	2 mg	232	128	55.17%

* “Both” includes transactions with only dispensed data or with all data (dispensed, administration, and waste). ** Eligible transactions include those with documented administration and/or waste. *** Half-wasted transactions are those where the documented waste was exactly half of the vial size.

**Table 2:** (**A**) Manual review of unverified waste: pharmacy WTW. (**B**) Summary of unverified waste manual review.

**(A)**		
**Medication**	**Sample Size (*n*)**	**Avg. Pharmacy WTW per Incident (min:sec)**
Fentanyl	193	6:54
Hydromorphone	57	7:12
Midazolam	26	4:44
Morphine	46	6:25
Ketamine	5	6:31
Lorazepam	6	6:48
**(B)**		
**Summary**		**Value**
Overall Average WTW per Incident (*n* = 333)		6:43 (min:sec)
Total Sample Size WTW (*n* = 333)		37:36:39 (hh:mm:ss)
Total Quarterly WTW (*n* = 4432)		496:08:16 (hh:mm:ss)
Annualized WTW		1984:33:04 (hh:mm:ss)

**Table 3:** (**A**) Incident-related transactions case review. (**B**) Pharmacy case review WTW.

**(A)**			
**Step Number**	**Description**	**Time**	**Standard Deviation**
1	Pull ADC Data	03:13 (min:s)	00:28 (sec)
2	Review Data	04:57 (min:s)	01:24 (min:s)
3	Provide Recommendation	3:20 (min:s)	00:26 (s)
Total	Time per Case	11:30 (min:s)	01:17 (min:s)
**(B)**			
**Summary**		**Time**	
Cases Reviewed (n = 35)		11:30 (min:s) per case	
Total Quarterly WTW (n = 170)		32:31:00 (hh:mm:ss)	
Annualized WTW		130:04:00 (hh:mm:ss)	

**Table 4:** IV push waste receptable costs.

Costs Related to Waste	Costs *
Set Up Cost for Med Station (Solid and Liquid)	USD 1750.00
Cost for Anesthesia Stations (Liquid only)	USD 800.00
Every six months, per replacement (Solid or Liquid Cartridge)	USD 125.00

* Costs are shown as list prices from 2022 for equipment, not including any discounts.

## Data Availability

The datasets presented in this article are not readily available due to institutional restrictions and the sensitive nature of the data, which pertain to controlled substance waste and utilization. These data are specific to internal operations and compliance protocols at the study institution and are not publicly shareable.

## Abbreviations

The following abbreviations are used in this manuscript:

ADC	Automated Dispensing Cabinet
Cath Lab	Cardiac Catheterization Laboratory
DEA	Drug Enforcement Agency
DRT	Diversion Response Team
ED	Emergency Department
EHR	Electronic Health Record
ICU	Intensive Care Unit
IRB	Institutional Review Board
IV	Intravenous
IVP	Intravenous Push
MAR	Medication Administration Record
NICU	Neonatal Intensive Care Unit
OR	Operating Room
PACU	Post-Anesthesia Care Unit
PICU	Pediatric Intensive Care Unit
PW	Product Waste
WTW	Workforce Time Waste
